# Supplementary material for: Gibberellin Positively Regulates Tomato Resistance to Tomato Yellow Leaf Curl Virus (TYLCV)
Source: Plants (Basel). 2024 May 6;13(9):1277. doi: 10.3390/plants13091277 (PMC11085062; doi:10.3390/plants13091277)
Supplement: Supplementary file 1 [file plants-13-01277-s001.zip › plants-2893625-supplementary.pdf]

Table S1. Sequence of the primers used in this work.

Primer	Sequence
ACTIN-F	GGAAAAGCTTGCCTATGTGG
ACTIN-R	CCTGCAGCTTCCATACC
TYLCV-Q-F	TAATCATTTCCACGCCCGTCTC
TYLCV-Q-R	CAGTATGCTTAATATCATCCCGTTGCTC
P450 94C1-F	CGCTTTCGATATCGTCAGTG
P450 94C1-R	CATGATGGGAAGTCCGCC
MYB117-F	ATGTCATCAACATCATCATCTTGTT
MYB117-R	TCAAGTGGCTCCTACTCCAA G
MYB20-F	ATGGGAAGGCAACCTTGTT
MYB20-R	TTAATTGTATTTTCTCAAAAGGAACAAA
MADS-MC-F	ATGGGAAGAGGAAAAGTTGAATTA
MADS-MC-R	TCATAGATGTTTATTCATGTTGTAAAGTG
ABA 3like-F	ATGGCATCAG AGCAAGAAGA
ABA 3like-R	TCAAACGTCGTTAACGCCT
MAPKKK 18-like-F	ATGATCTGGAAAAAGCTTAAGGT
MAPKKK 18-like-R	TTATCCTCTGAAGTTGCTGTCAC
PR5-F	ATGAGTCACTTGACAACTTGTTTAGT
PR5-R	TTAAGCAACTTCAAGAGTACTTGTAG
Wound-induced inhibitior-F	ATGGAGTCAAAGTTTGCTCAC
Wound-induced inhibitior-R	TTAAGTCACCACAGGCATTTGTA
